# P-819. Epidemiology of *Serratia marcescens* Bloodstream Infections in a Hospital System in the Southeast U.S

**DOI:** 10.1093/ofid/ofae631.1011

**Published:** 2025-01-29

**Authors:** Marilyn Niravath, Dierdre B Axell-House

**Affiliations:** Houston Methodist, Houston, Texas; Houston Methodist, Houston, Texas

## Abstract

**Background:**

*Serratia marcescens* (*Sm*) is an environmental gram-negative bacilli that can cause a variety of serious human infections. *Sm* is frequently attributed as a hospital acquired infection. However, there is not extensive documentation as to the common etiologies of *Serratia* bloodstream infections (BSI), frequency of antibiotic resistance, and risk factors for recurrent infection or mortality.

**Methods:**

We conducted a retrospective chart review of a cohort of patients (pts) ≥ 18 years old with *Serratia marcescens* BSI from 5/2016 to 10/2022 at our hospital system. *Sm* BSI was defined as ≥ 1 blood culture positive for *Sm*. Multidrug resistance was defined according to Centers for Disease Control (CDC) criteria. Patients were assessed for outcomes of recurrence of bacteremia (positive blood culture with same isolate within 3 months of clearance) and 30-day mortality.

**Results:**

A total of 243 patients had *Sm* BSI during the study period. The most frequent etiology was central line-associated BSI (38%) followed by skin and soft tissue infection (12%). The majority (96%) of isolates were not multidrug resistant. Most patients had a repeat blood culture (67%), and of these patients, 15% had recurrence of bacteremia. A total of 44 patients (18%) died within 30 days. Echocardiogram was performed in 60% of our patients, with 7 patients diagnosed as having endocarditis or device endocarditis. Current active or history of injection drug use occurred in 7.5% of patients.

**Conclusion:**

Our retrospective study demonstrates a low burden of antibiotic resistance in *Sm* at our hospital system. Similar to prior studies, CLABSI is a frequent cause of *Sm* BSI, and foreign materials/devices are common in patients with *Sm* BSI. Our patient population has a lower rate of IVDU compared to other studies of *Sm* BSI, and endocarditis is not common. Further analysis is needed to evaluate treatment regimens and other risk factors for recurrent BSI and mortality.

**Disclosures:**

**All Authors**: No reported disclosures

